# Necrosis-inducing peptide has the beneficial effect on killing tumor cells through neuropilin (NRP-1) targeting

**DOI:** 10.18632/oncotarget.8719

**Published:** 2016-04-13

**Authors:** Ji-Young Kim, Ji-Hae Han, Geon Park, Young-Woo Seo, Cheol-Won Yun, Byung-Chul Lee, Jeehyeon Bae, Ae Ran Moon, Tae-Hyoung Kim

**Affiliations:** ^1^ Department of Biochemistry, Chosun University School of Medicine, Dong-Gu, Gwang-Ju, Korea; ^2^ Department of Laboratory Medicine, Chosun University School of Medicine, Dong-Gu, Gwang-Ju, Korea; ^3^ Korea Basic Science Institute Gwang-Ju Center, Chonnam National University, Buk-Gu, Gwang-Ju, Korea; ^4^ School of Life Science and Biotechnology, Korea University, Seoul, Korea; ^5^ Genoflux. Co., Ltd., Sejong City, Korea; ^6^ School of Pharmacy, Chung-Ang University, Dongjak-Gu, Seoul, Korea

**Keywords:** necrosis, pro-necrotic peptide, mitochondrial targeting domain, NRP-1, Noxa

## Abstract

The therapeutic efficacy of most anti-cancer drugs depends on their apoptosis-inducing abilities. Previously, we showed that a peptide containing the mitochondrial targeting domain (MTD) found in Noxa, a BH-3 only protein of Bcl-2 family, induces necrosis. Here, a fusion peptide of neuropilin-1 (NRP-1) targeting peptide and MTD peptide, designated tumor homing motif 17:MTD (TU17:MTD), was found to induce necrosis in cancer cells *in vitro* and to cause the regression of tumors when intravenously injected into mice bearing subcutaneous CT26 colorectal carcinoma tumors. The necrosis within tumor tissues was evident upon administering TU17:MTD. TU17:MTD penetrated into tumor cells by targeting to Neuropilin-1, which could be blocked by anti-NRP-1 antibody. The efficacy of TU17:MTD on tumor regression was higher than that of TU17:_D_(KLAKLAK)_2_, a fusion peptide of NRP-1 targeting peptide and a pro-apoptotic peptide. The necrotic cell death within tumor tissues was evident at day 1 after administering TU17:MTD systemically. Transplanted subcutaneous substantially reduced in size within two weeks and 5 days, respectively, with no apparent side effects. Together, these results propose that the pro-necrotic peptide MTD may present an alternative approach for development of targeted anti-cancer agents.

## INTRODUCTION

Over the last three decades, studies on apoptosis have revealed that the signaling pathways of apoptosis are very complicated involving various mediators such as caspases, Bcl-2 family proteins, and IAP proteins [1, 2]. Most anti-cancer drugs, including chemotherapeutic agents, such as, cisplatin, etoposide, and paclitaxel, and targeted anti-cancer drugs, such as, imatinib induce apoptotic cell death. However, many tumor cells develop escape mechanisms that involve the deletion or modulation of key mediators of apoptosis, and eventually develop a resistance to anti-cancer drugs. This emergence of tumor resistance restricts the therapeutic efficacies of pro-apoptotic anti-cancer drugs, and is one of the major causes of relapse after anti-cancer drug treatment [3]. The other unfavorable aspect of pro-apoptotic anti-cancer drugs is that they suppress or deplete subgroups of cells in the immune system, especially T-cells [3]. This is mainly due to their direct toxic effects on immune cells [4, 5]. Pro-apoptotic anti-cancer drugs can trigger minimal immunogenic reactions because apoptosis is a type of cell death that minimizes the release of intracellular danger signals into the surrounding environment, thereby, ameliorating inflammatory immune reactions [1, 2]. Although a large number of studies have revealed the detailed signaling pathways involved in the apoptotic mechanism, at the biochemical and genetic levels, including the role of caspases, Bcl-2 family proteins, and IAP proteins [1, 2], strategies to overcome the limitations of pro-apoptotic anti-cancer drugs are still not established yet.

In contrast to apoptosis, necrosis, which is characterized by the swelling of subcellular organelles and a loss of cytoplasmic membrane integrity, release of intracellular danger signals into surrounding environments and the activation of immune responses, was disregarded as a passive and harmful form of cell death for several decades. Recent evidence, however, indicates that necrosis is a regulated form of cell death. Some apoptosis-associated genes like receptor-associated protein (RIP)1 and RIP3 have been shown to be involved in the regulated necrosis or necroptosis [6–8]. RIP3 phosphorylates the mixed lineage kinase-like protein (MLKL), resulting in membrane localization of MLKL by releasing four-helix bundle domain of MLKL, which then causes membrane disruption and finally necroptosis [9–12]. However, the key mediators of necrosis remain largely unknown.

We reported a 10-residue long, pro-necrotic peptide derived from the mitochondrial targeting domain (MTD) located in the C-terminal region of Noxa, a BH-3 only protein of Bcl-2 family [13]. The MTD region of Noxa is responsible not only for mitochondrial targeting of Noxa [14] but also for mitochondrial fragmentation induced by Noxa [15]. Two leucine residues (L45 and L49) in the region of MTD play a key role in mitochondrial fragmentation induced by Noxa [15]. Moreover, we showed that the MTD peptide conjugated with a cell penetration peptide like eight arginine (R8:MTD) potently induces necrosis in many tumor cells via an abrupt spike in cytoplasmic calcium release from mitochondria. The two leucine residues of the MTD that played a key role in mitochondrial fragmentation are also key residues in induction of necrosis by R8:MTD [13]. R8:MTD peptide induced necrosis in various tumor cells *in vitro* within 10 ~ 30 minutes in a caspase-independent manner. Although the molecular mechanisms of R8:MTD-induced necrosis are largely unknown, it may directly damage mitochondria, rather than activating a cell death signaling cascade [13].

Here, we describe a novel pro-necrotic peptide anti-cancer agent based on the combination of MTD with tumor-homing motifs, and suggest that pro-necrotic agents such as MTD may be an alternative way to overcome the limitations of pro-apoptotic anti-cancer drugs.

## RESULTS

### TU17:MTD, a peptide containing MTD, kills tumor cells

**Figure 1:**
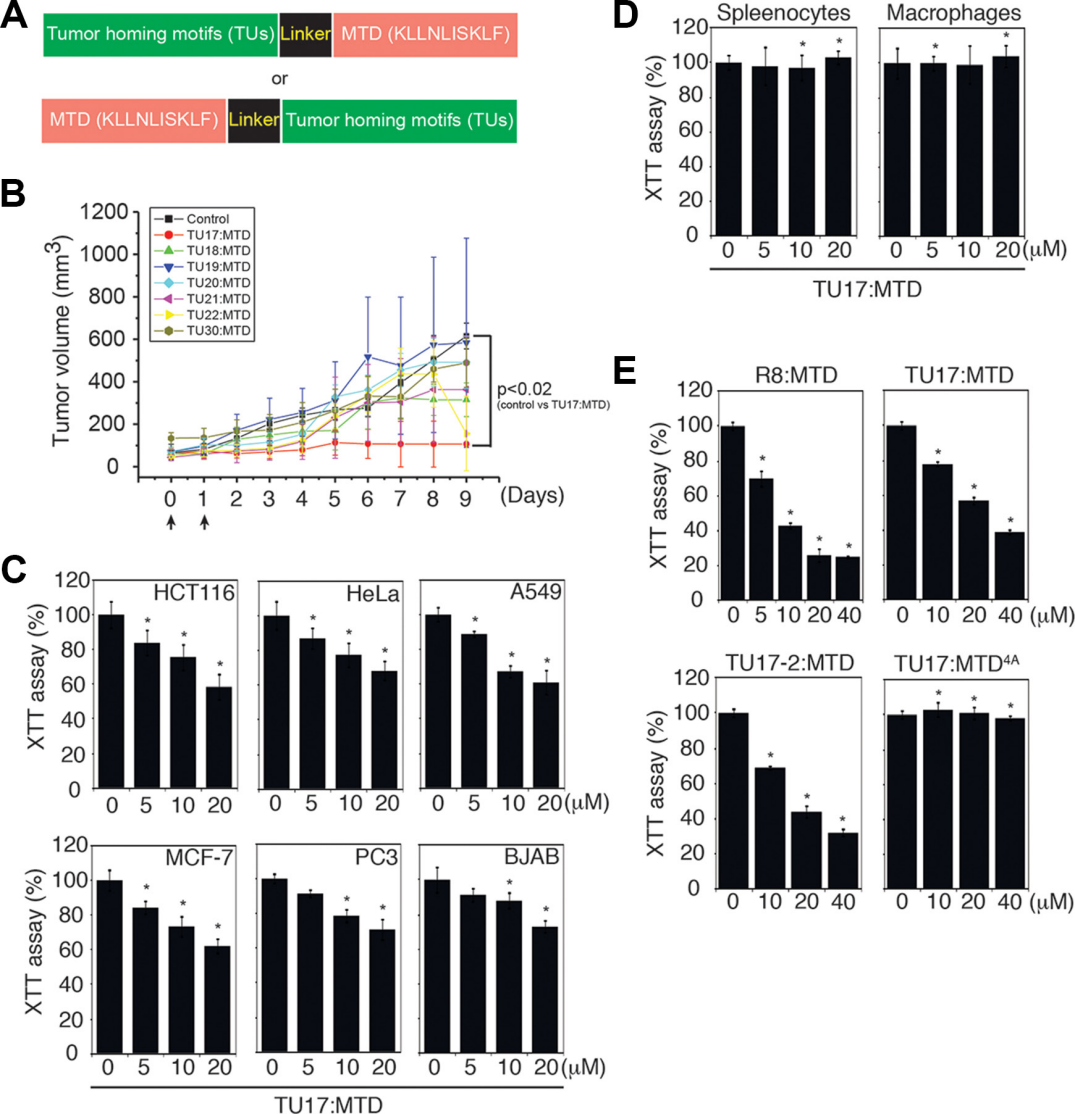
Identification of TU17:MTD as a tumor targeting peptide (**A**) Schematic diagram of the fusion peptides consisting of the tumor-homing motif, linker, and mitochondrial targeting domain (MTD). (**B**) Tumors were generated over 2 weeks after s.c. injection of CT26 cells into the backs of BALB/c mice. When tumor volumes reached palpable size, the indicated TU:MTDs (100 μl of 1 mM TU:MTDs) or PBS were administrated i.v. twice or three times. Tumor dimensions in the TU:MTDs-treated groups (*n* = 3) and PBS-treated group (*n* = 3) were measured using a caliper and tumor volumes were calculated at the indicated days using longest diameter × width^2^ × 0.5. Arrows indicate the points of peptide injection. *P* values < 0.02 (control group vs TU17:MTD at day 5, 6, 7, 8, and 9) (**C**) HCT116, HeLa, A549, MCF-7, PC3, and BJAB cells were treated with TU17:MTD (0 ~ 20 μM) for 30 minutes, and cell viability was monitored using XTT assays. *P < 0.05 (**D**) Primary splenocytes and macrophages were treated with TU17:MTD (0 ~ 20 μM) for 30 minutes, and cell viability was monitored by XTT assay. *P < 0.05 (**E**) CT26 cells were treated with R8:MTD, TU17:MTD, TU17-2:MTD or TU17:MTD^4A^ (0 ~ 40 μM) for 4 hours, and cell viability was monitored by XTT assay. Results in C to E are expressed as means ± SD (triplicates), and are representative of at least two independent experiments. *P* values are for experimental groups versus controls and were calculated using Dunnett's *t*-test. *P < 0.05.

To design a MTD peptide anti-cancer drug, the MTD peptide was fused to various known tumor-homing motifs through its N-terminal or C-terminal region [16], and a linker was introduced between these two motifs to impart flexibility and minimize steric hindrance (Figure 1A, Supplementary Table S1). The MTD peptides fused with tumor-homing motifs (hereafter designated TU:MTDs) were synthesized as linear or cyclic entities using L-amino acids (Supplementary Table S1), and were evaluated for their killing activity using CT26 cells *in vitro* (Supplementary Figure S1). TU2, 3, 11, 15 ~ 22:MTD induced the typical morphological features of necrosis. When injected into BALB/c mice (20 gm), R8:MTD (25 μl ~ 50 μl of 1 mM R8:MTD/mouse) was found to be lethal (data not shown), showing that the tumor targeting specificity of TU:MTDs is a major concern. Thus, BALB/c mouse movements were also evaluated within 30 minutes of the intravenous injection of a single dose of 75 μl of 1 mM TU:MTDs per mouse. It was found that TU8:MTD is highly toxic although it was not cytotoxic to CT26 cells *in vitro* (Supplementary Table S2). While many TU:MTDs (1, 4, 10, 11, 15, 18, and 21) appeared to be toxic, as determined by observing the slow movements of the mice within 30 minutes of administration, other TU:MTDs (2, 3, 5, 6, 7, 9, 16, 17, 19, 20, and 22) showed no apparent toxicities up to one week after administration (Supplementary Table S2). We also searched for a TU:MTD with a potent *in vivo* effect by observing tumor volumes in three BALB/c mice bearing CT26 adenocarcinoma that were injected with 100 μl of 1 mM TU:MTDs per day for 2 or 3 consecutive days (Figure 1B). Some TU:MTDs were found to suppress tumor growth, but not to reduce tumor sizes. TU17:MTD was found to have a stronger suppressive effect on tumor growth than did the other TU:MTDs (Figure 1B). The tumor-homing motif of TU17:MTD has a “RPARPAR” sequence containing the C-end rule (CendR) element that has known to bind to neuropilin-1 (NRP-1) [17, 18], although the “RPARPAR” sequence is located at the N-terminus of the MTD rather than at the C-terminus. Thus, we further tested the effects of TU17:MTD on tumor growth *in vitro* and *in vivo*.

When developing anti-cancer drugs, minimization of the adverse effects on normal cells is one of the major concerns. Therefore, we determined whether TU17:MTD could discriminate tumor and normal cells *in vitro*. When TU17:MTD was added to HeLa (cervix adenocarcinoma), HCT116 (colorectal carcinoma), MCF-7 (breast adenocarcinoma), A549 (lung carcinoma), PC3 (prostate adenocarcinoma), or BJAB (B cell lymphoma) cells, it reduced cell viability in a concentration-dependent manner, as measured by XTT assay (Figure 1C). In contrast, the same concentrations of TU17:MTD did not induce cell death in normal peritoneal macrophages or splenocytes (Figure 1D). TU17:MTD and TU17-2:MTD, a second form of TU17:MTD in which the linker sequence GG is replaced by GFLG, presented a reduced killing activity compared to that of R8:MTD. We didn't observe any significant differences between TU17:MTD and TU17-2:MTD in terms of *in vitro* killing activity, suggesting that replacement of GG by GFLG has no advantages. Previously, we have shown that replacement of four leucine residues in MTD (K*LL*N*L*ISK*L*F) to alanine, indicated as MTD^4A^ (K*AA*N*A*ISK*A*F), abolishes the killing activity and mitochondrial targeting activity of MTD [13]. As expected, TU17:MTD^4A^ abrogated its killing activity (Figure 1E), indicating that the killing activity of TU17:MTD is caused by MTD but not by tumor homing motif (RPARPAR) or linker sequence (GG).

### TU17:MTD, but not TU17:_D_(KLAKLAK)_2_, induces necrosis in tumor cells

**Figure 2:**
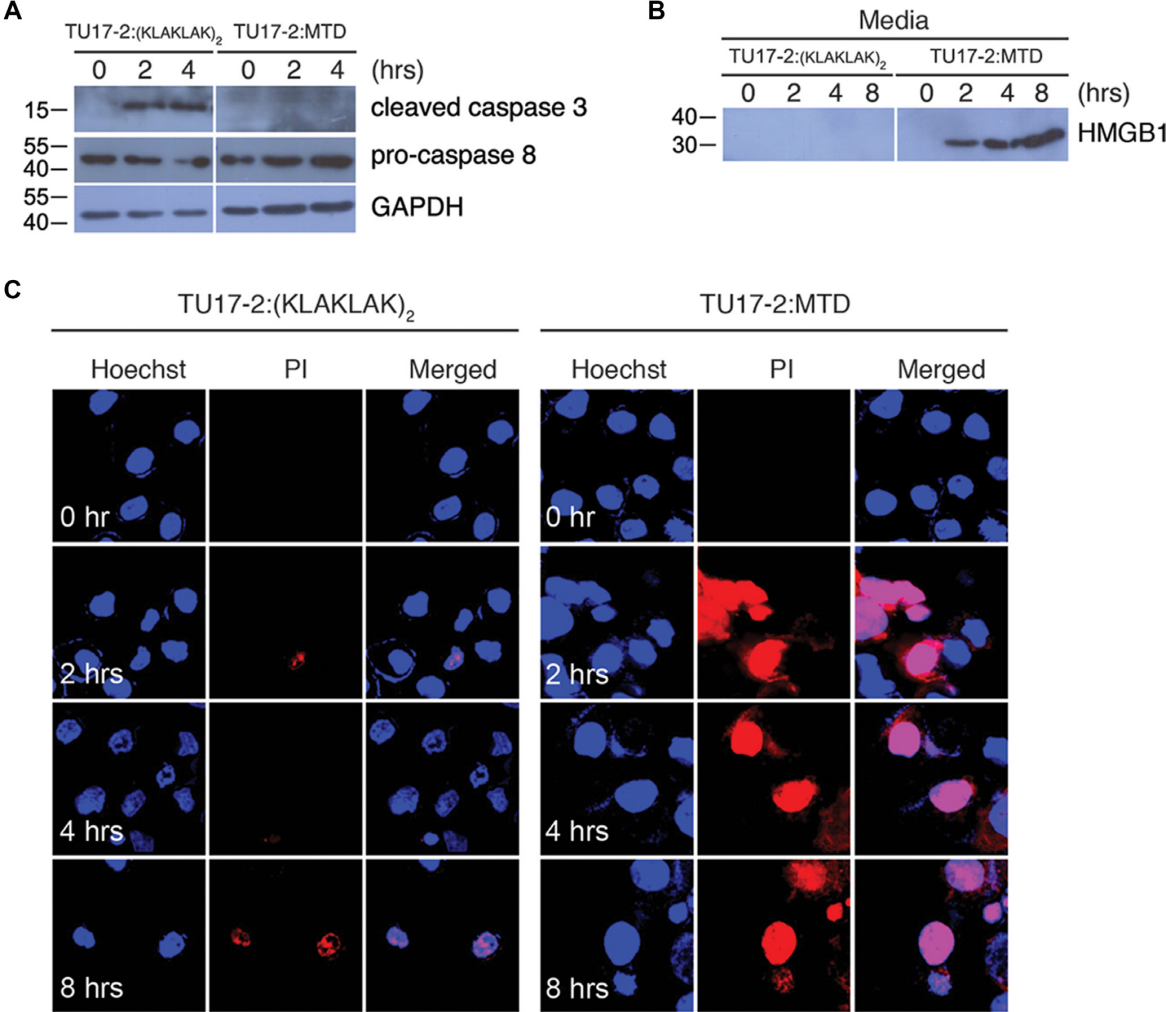
TU17-2:MTD induces necrosis, whereas TU17-2:_D_(KLAKLAK)_2_ induces apoptosis (**A**) Lysates of CT26 cells treated with TU17-2:_D_(KLAKLAK)_2_ (80 μM) or TU17-2:MTD (20 μM) were subjected to western blot with anti-caspase-3 (1:1000), anti-pro-caspase-8 (1:500) or anti-GAPDH (1:2000) antibodies. (**B**) Culture media from cells treated with TU17-2:_D_(KLAKLAK)_2_ (80 μM) or TU17-2:MTD (20 μM) were precipitated with TCA. Samples were subjected to western blot analysis by using an anti-HMGB1 (1:1000) antibody. (**C**) CT26 cells were treated with TU17-2:_D_(KLAKLAK)_2_ (80 μM) or TU17-2:MTD (20 μM), and were stained with Hoechst/PI. Images were obtained using an Olympus confocal microscope.

To examine whether TU17-2:MTD induces necrosis or apoptosis in tumor cells, we observed the morphological and biochemical features of apoptosis and necrosis in CT26 cells treated with TU17-2:MTD or TU17:_D_(KLAKLAK)_2_. We used _D_(KLAKLAK)_2_ peptide, an well-known apoptosis-inducing peptide, to design TU17:_D_(KLAKLAK)_2_ [19–22]. To show that TU17:_D_(KLAKLAK)_2_ causes apoptosis in CT26 cells, activation of caspase-3 and caspase-8, which is a biochemical indicator of apoptosis, was examined by western blot. The results showed that TU17:_D_(KLAKLAK)_2_ but not TU17-2:MTD could activate caspase-3 and caspase-8 (Figure 2A). On the other hand, CT26 cells treated with TU17-2:MTD, but not with TU17:_D_(KLAKLAK)_2_, released HMGB1, a biochemical indicator of necrosis, into culture medium *in vitro* (Figure 2B). Morphological changes of the nucleus and cell membrane permeabilization in response to TU17:_D_(KLAKLAK)_2_ or TU17-2:MTD were further observed to distinguish the modes of cell death. Permeabilization of cell membrane, a morphological indicator of necrosis, analyzed by PI-staining was observed mostly in CT26 cells treated with TU17-2:MTD but not in cells treated with TU17:_D_(KLAKLAK)_2_ (Figure 2C, and Supplementary Figure S2A). Condensed nuclei, a morphological indicator of apoptosis, analyzed by Hoechst staining were observed mostly in CT26 cells treated with TU17:_D_(KLAKLAK)_2_ but not in cells treated with TU17-2:MTD (Figure 2C, and Supplementary Figure S2B). These results indicated that TU17-2:MTD causes necrosis, whereas TU17:_D_(KLAKLAK)_2_ causes apoptosis.

### TU17:MTD regresses tumor volume in mice

**Figure 3:**
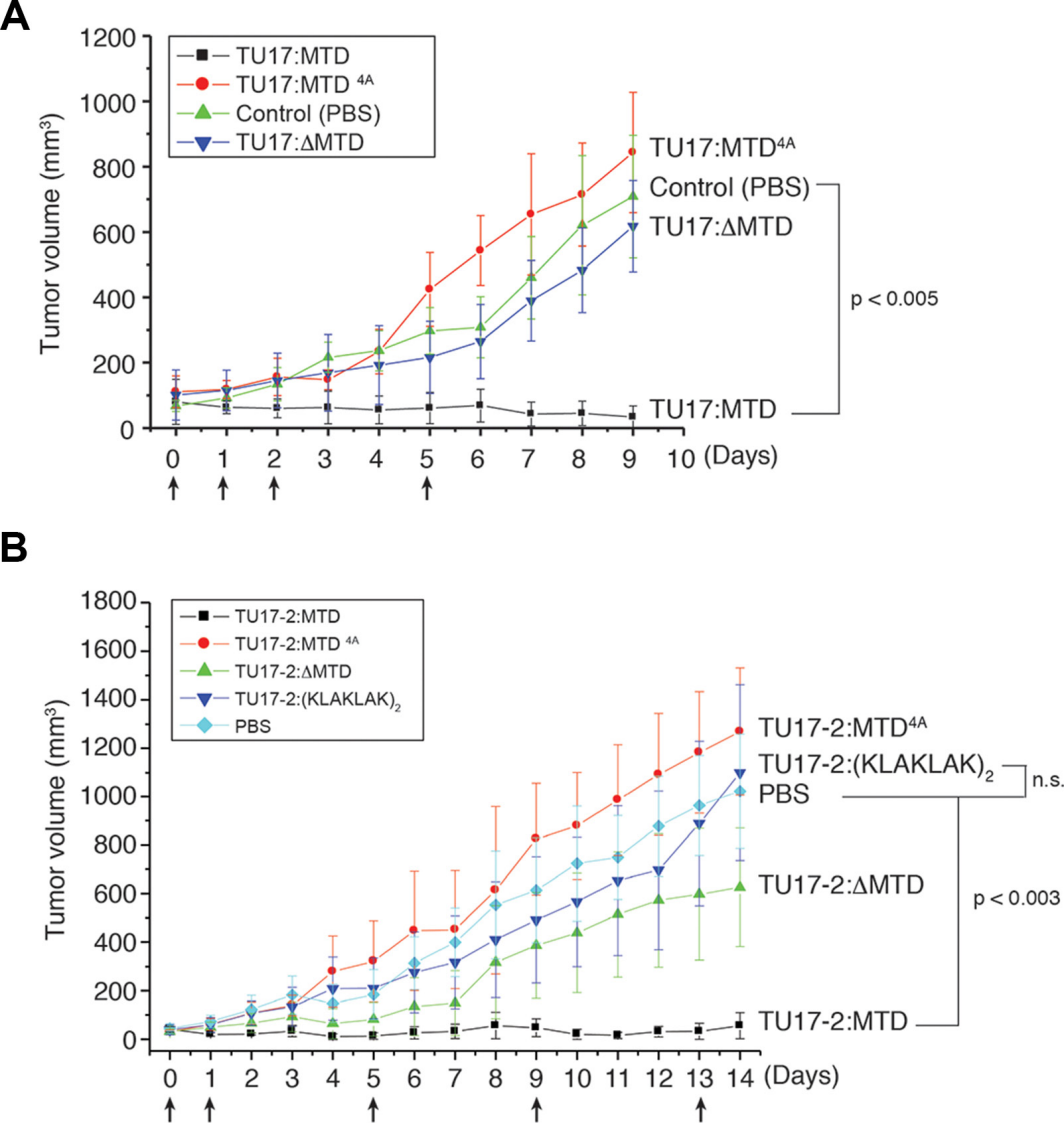
Effects of TU17:MTD, TU17-2:MTD, and TU17-2:_D_(KLAKLAK)_2_ on tumor tissues *in vivo* (**A**) Tumors were generated over 7~8 days after s.c. injection of CT26 cells into the backs of BALB/c mice. TU17:MTD, TU17:MTD^4A^, TU17:ΔMTD (200 μg/mouse/day, total volume: 100 μl) or PBS (100 μl) were administrated i.v. once daily as indicated (arrows). Tumor dimensions in mice from the TU17:MTD-treated group (*n* = 10), PBS-treated group (*n* = 10), TU17:MTD^4A^–treated group (*n* = 10), and TU17:ΔMTD-treated group (*n* = 10) were measured using a caliper and tumor volumes were calculated. Initial tumor size at day 0 was 70 ± 20 mm^3^. Arrows indicate the points of peptide injection. *P* < 0.005 (PBS vs TU17:MTD) (**B**) Mice bearing CT26 tumors were i.v. administrated with TU17-2:MTD (*n* = 6), TU17-2:_D_(KLAKLAK)_2_ (*n* = 6), TU17-2:MTD^4A^ (*n* = 6), TU17-2:ΔMTD (*n* = 6), or PBS (*n* = 6) once daily as indicated (arrows). Initial tumor size at day 0 was 40 ± 10 mm^3^. Arrows indicate the points of peptide injection. *P* < 0.003 (PBS vs TU17-2:MTD), n.s. (non-significant).

To test the efficacy of TU17:MTD in tumor killing *in vivo*, we first generated subcutaneous CT26 cell tumors in BALB/c mice and started treatment when the tumors reached a palpable size. TU17:MTD or TU17-2:MTD (100 μl of 1 mM peptides/mouse/day) was intravenously administrated into the mice via tail vein. Tumor growth was greatly suppressed within 9 days, whereas control animals, which were given saline, exhibited continuous tumor growth (Figure 3A). In contrast, the administration of TU17:ΔMTD or TU17-2:ΔMTD (tumor-homing motif without MTD) or TU17:MTD^4A^ resulted in progressive tumor growth (Figure 3A), showing that TU17:MTD and TU17-2:MTD showed comparable anti-tumor activity. In addition, we tested the extent of tumor volume reduction in response to TU17-2:MTD or TU17-2:_D_(KLAKLAK)_2_. TU17-2:MTD induced a significant reduction of the tumor volume, whereas TU17-2:_D_(KLAKLAK)_2_ showed no reduction of tumor volume, similar to the results obtained with treatment of TU17:MTD^4A^or PBS (Figure 3B). These results suggest that the necrosis-inducing activity of MTD, instead of apoptosis-inducing agents, may present a beneficial potential in anti-cancer drug development.

**Figure 4:**
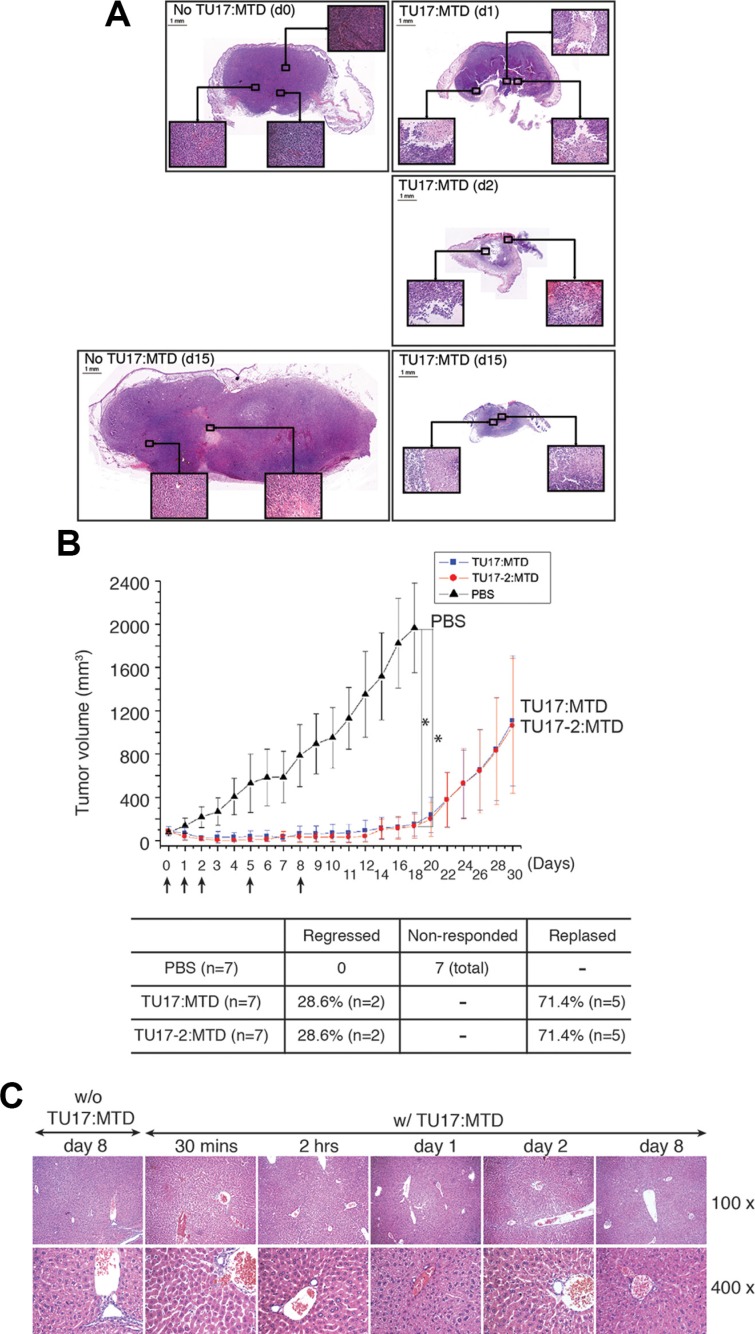
TU17:MTD induces necrosis in tumor tissues (**A**) Tumor tissues were obtained at day 1 (d1) post-TU17:MTD injection (a single injection), and on day 2 (d2) and day 15 (d15) from TU17:MTD-treated group. Tumor tissues were obtained at day 0 (d0) or day 15 (d15) from the PBS-treated group. Images of H&E stained sections (Magnification, 40 ×) covering the whole tumor tissues were taken under a light microscope, and photo-merged using Adobe Photoshop CS5. Inset magnification 200 ×. (**B**) Tumors were generated as described above using CT26 cells. TU17:MTD, TU17-2:MTD (200 μg/mouse/day, total volume: 100 μl) or PBS (100 μl) were administrated i.v. into the mice once daily as indicated (arrows). Tumor volumes in mice from the TU17:MTD-treated group (*n* = 7), TU17-2:MTD group (*n* = 7), or PBS-treated group (*n* = 7) were measured for 30 days. Initial tumor size at day 0 was 70 ± 20 mm^3^. *P values < 0.005 (PBS vs TU17:MTD or TU17-2:MTD at each) (**C**) Liver tissues, obtained from the TU17:MTD-treated group at the indicated time points or from the PBS-treated group at day 0 as a control, were stained with H&E, and images (100 ×, 400 ×) were taken under a light microscope.

To further investigate the changes in tumor tissues after the administration of TU17:MTD, tumor tissues were stained with H&E at 1, 2, and 15 days after TU17:MTD treatment was initiated. While tumor tissues in control mice were densely filled with tumor cells, massive necrotic cell death was observed in the tumor tissues of TU17:MTD-treated mice (Figure 4A). Necrotic cell death within tumors were evidently observed as early as 30 minutes after TU17:MTD treatment (Supplementary Figure S3). A significant reduction in tumor volume was evident at day 1 and 2 after injection. At day 15, tumors had greatly reduced in TU17:MTD-treated mice, but remained very large in control mice (Figure 4A).

To evaluate the tumor-killing effects of TU17:MTD and TU17-2:MTD on tumor *in vivo* over extended time periods, we treated the animals with the peptides for up to 8 days after tumor was generated as shown in Figure 3. After completing the treatment, we observed for 30 days to assess whether the tumors would relapse or not. As expected, the tumors were regressed while the peptides were injected (total animal number of each group = 7). Upon completing the treatments, some mice (approximately 28%) presented a complete regression of the tumors, whereas others (approximately 71%) showed relapsed tumor growth (Figure 4B).

To assess liver toxicity induced by TU17:MTD in mice, microscopic analysis of liver tissue samples from TU17:MTD-treated mice showed no apparent damages (Figure 4C). Serum levels of aspartate aminotransferase (AST) and alanine aminotransferase (ALT) were substantially higher in CCl_4_-treated mice, a chemically-induced liver damage model [23], but normal in TU17:MTD-treated mice (Supplementary Table S3). Additionally, the analysis of complete blood count in TU17:MTD-treated mice showed no significant cytotoxicity on peripheral blood cells (Supplementary Table S4). These results support the notion that TU17:MTD does not cause severe damages on liver and peripheral blood cells in mice.

### Anti-NRP-1 antibody blocks the entrance of TU17:MTD into CT26 cells and cell death induced by TU17:MTD

**Figure 5:**
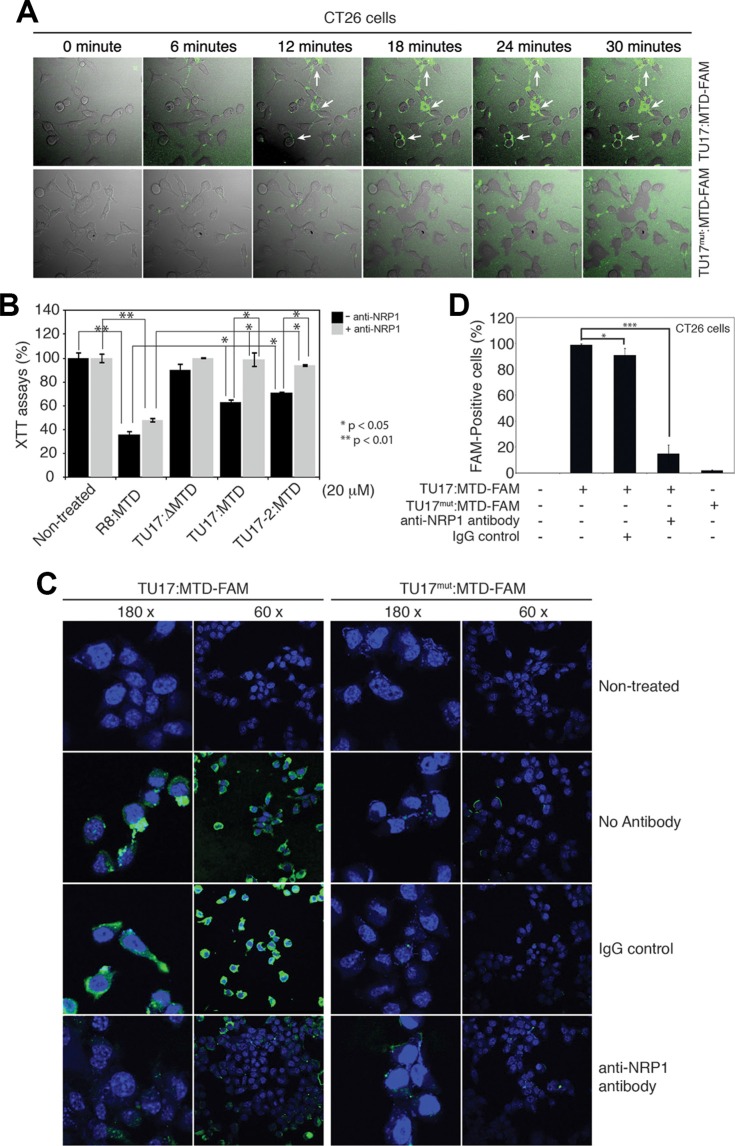
Anti-NRP-1 antibody blocks TU17:MTD-induced necrosis (**A**) CT26 cells (2 × 10^4^ cells/cm^2^) were treated with TU17:MTD-FAM (500 nM) peptide or TU17^mut^:MTD (500 nM) peptide. Live cell images (magnification 60 ×) were obtained at 6-minute intervals for 30 minutes using an Olympus confocal microscope. (**B**) CT26 cells were treated with the indicated MTD peptides in the presence or absence of anti-NRP-1 antibody (1:100 dilution, pretreatment) for 1 hour. The viability was measured by using the XTT assay. *P < 0.05, **P < 0.01. (**C**) CT26 cells were treated with TU17:MTD-FAM or TU17^mut^:MTD-FAM in the presence or absence of the anti-NRP-1 antibody (1:100 dilution, pretreatment). At two hours after treatment, the samples were counterstained with the ProLong Gold containing DAPI, and were observed using Olympus confocal microscope. (**D**) CT26 cells (2 × 10^4^ cells/cm^2^) were treated with TU17:MTD-FAM (500 nM) peptide or TU17^mut^:MTD (500 nM) peptide in the presence or absence of anti-NRP-1 antibody or IgG control as described in Figure 5C. Images were captured at 30 minutes after peptide treatment by using an Olympus confocal microscope. The percentage of FAM-positive cells (%) were determined (*n* = over 200 cells). *P < 0.05, ***P < 0.001.

To identify the detailed mechanism by which TU17:MTD can specifically target the tumor cells, we first tested whether TU17:MTD conjugated with FAM at its C-terminal end (TU17:MTD-FAM) or TU17^mut^:MTD-FAM, in which RPARPAR of TU17 is replaced by APAAPAA in TU17:MTD-FAM, can penetrate into tumor cells *in vitro*. The penetration of TU17:MTD-FAM into CT26 cells or HeLa cells was initially observed within 6 ~ 12 minutes of the peptide treatment. Thirty minutes after the treatment, TU17:MTD-FAM was observed within most of the CT26 cells and HeLa cells (Figure 5A and Supplementary Figure S4A, respectively); however, TU17^mut^:MTD-FAM could not penetrate into CT26 cells and HeLa cells (Figure 5C–5D and Supplementary Figure S4B–S4C, respectively).

The targeting specificity of TU17:MTD to tumor cells is likely to be determined by the “RPARPAR” sequence that is previously known to bind to NRP-1, a co-receptor for vascular endothelial growth factor (VEGF), expressed in tumor vessels and in most carcinomas [24–26]. Thus, we tested whether an anti-NRP-1 antibody could inhibit the tumor targeting and killing activity of TU17:MTD or not. Cell death that was induced by TU17:MTD or TU17-2:MTD alone in CT26 cells was inhibited by the anti-NRP-1 antibody; however, R8:MTD-induced cell death could occurred irrespective of the presence of the anti-NRP-1 antibody. Moreover, TU17:ΔMTD could not induce the cell death irrespective of the presence of the anti-NRP-1 antibody (Figure 5B). Furthermore, the anti-NRP-1 antibody inhibited the penetration of TU17:MTD-FAM into CT26 cells and HeLa cells (Figure 5C and Supplementary Figure S4B, respectively) *in vitro*, and the number of FAM-positive cells was substantially decreased by the anti-NRP-1 antibody in CT26 cells (Figure 5D) and HeLa cells (Supplementary Figure S4C). These results indicate that TU17:MTD penetrates into tumor cells through NRP-1 and its killing activity depends on the penetration of cell membrane.

### Anti-NRP-1 antibody blocks TU17:MTD-induced tumor regression

**Figure 6:**
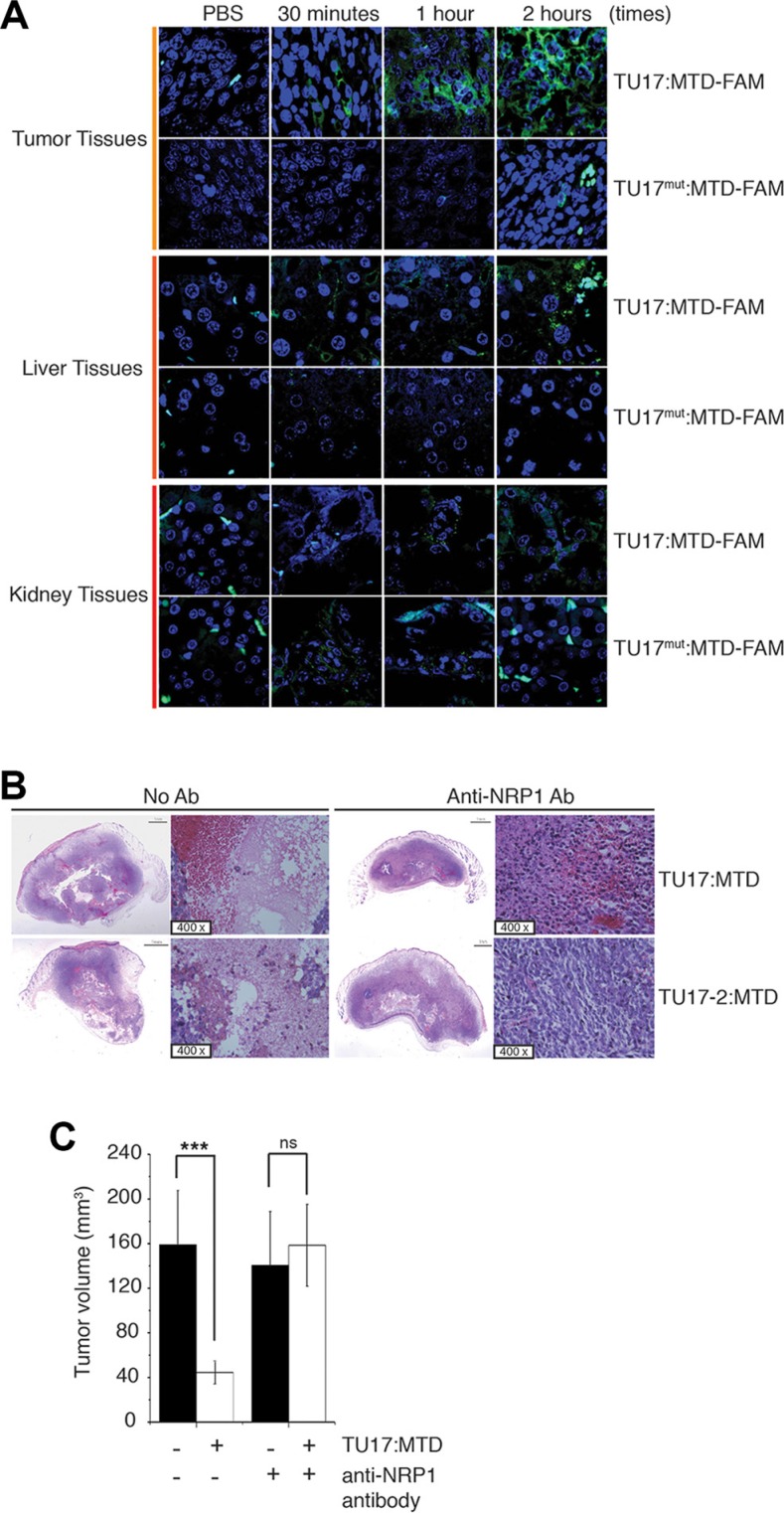
Anti-NRP-1 antibody blocks necrosis in tumor induced by TU17:MTD (**A**) TU17:MTD-FAM, TU17^mut^:MTD-FAM (100 μl of 1 mM), or PBS was i.v. injected into the mice bearing tumors generated with CT26 cells. Mice were sacrificed at 30 minutes, 1 hour, and 2 hours after injection. Tissues from the tumor, liver, and kidney were processed for paraffin remove with Histoclear solution. The sections were counterstained with ProLong Gold containing DAPI. Images (magnification 400 ×) were obtained using Olympus confocal microscope. (**B**) The anti-NRP-1 antibody (1:100, *n* = 5) or PBS (*n* = 5) was i.v. injected into the mice bearing the tumor at 2 hours before TU17:MTD administration. Mice were sacrificed at day 1 after treatments. The samples were stained with H&E. (**C**) The mice bearing CT26 cell tumor were i.v. injected with an anti-NRP-1 antibody (1:100, *n* = 5) or PBS (*n* = 5) at 2 hours before TU17:MTD administration. Mice were sacrificed at 1 day after treatments. Tumor volumes were measured. ***P < 0.001, ns (non-significant).

To further examine the targeting of TU17:MTD to tumor cells *in vivo*, TU17:MTD-FAM were injected into mice bearing tumor. Within 1 ~ 2 hours of treatment, the penetration of TU17:MTD-FAM was observed mainly in tumor cells but sparsely in the liver or kidney cells *in vivo*, whereas TU17^mut^:MTD-FAM was sparsely observed in tumor, liver or kidney cells (Figure 6A). Furthermore, the tumor targeting of TU17:MTD-FAM was blocked by TU17:ΔMTD (RPARPAR alone without MTD) *in vivo* (Supplementary Figure S5A). TU17:ΔMTD will compete with TU17:MTD-FAM for NRP-1 targeting, resulting in reduction of TU17:MTD-FAM targeting to NRP-1 by TU17:ΔMTD. Indeed, total radiant efficiency measured by optical imaging in tumor tissues treated with TU17:MTD-FAM alone was greatly reduced by co-treatment with TU17:ΔMTD, although the fluorescence intensity in the blood of mice treated with TU17:MTD-FAM alone was compatible with that in the blood of mice treated with TU17:MTD-FAM and TU17:ΔMTD (Supplementary Figure S5B). These results indicate that the targeting specificity and penetration ability of TU17:MTD into tumor cells *in vivo* depend on the sequence of TU17 part but not MTD part.

The killing activity of TU17:MTD or TU17-2:MTD on tumor tissues was substantially inhibited when the anti-NRP-1 antibody was pre-injected into tumor-bearing mice *in vivo* (Figure 6B), and the extent to which TU17:MTD or TU17-2:MTD injection reduced tumor volume at day 1 was significantly decreased (Figure 6C). Together, these results indicate that TU17:MTD and TU17-2:MTD target, penetrate, and kill the tumor cells by targeting to NRP-1 on tumor cells.

## DISCUSSION

Strategically, the action of peptide-based anti-cancer drugs depends on two functional motifs, a tumor-homing motif and a tumor-cell killing motif. If the tumor-cell killing motif is functional inside of the cells, the tumor-homing motif should satisfy both tumor specific targeting and penetration of cargo through the cell membranes of tumor cells.

Although several studies have identified tumor-homing motifs that satisfy these conditions, tumor-homing motifs conjugated with a pro-apoptotic peptide, for example, _D_(KLAKLAK)_2_ [21] as shown in Figure 3, or with a chemotherapeutic agent [27–30] have shown limited anti-tumor efficacy, possibly due to the poor killing activities of pro-apoptotic motifs, short circulating half-life, and/or tumor cell resistance. The serum stability of peptide-based drugs without modifications are, in general, less than 1 hour [31–34]. For instance, the half-life of glucagon-like protein-1 (GLP-1) is less than 5 minutes because dipeptidyl peptidase-4 (DPP-4) rapidly inactivates GLP-1 peptide in the circulation [32, 35]. Thus, the increased half-life of peptide-based drug in the circulation is necessary to achieve the sufficient biological efficacy of the peptide *in vivo*. In addition, most pro-apoptotic molecules need at least several hours or even several days to induce apoptosis. Therefore, long-lasting peptide-based pro-apoptotic anti-cancer drugs are required to provide sufficient anti-cancer efficacy. Indeed, recent several reports showed that a long-lasting peptide conjugated to paclitaxel has the greatly improved efficacy on tumor growth [31, 36]. Thus, many efforts strived to design and search for the long-lasting peptide by modifying amino acid residues with unnatural residues or by conjugating AG10, fatty acids or albumin [37–40]. In view of this perspective, TU17:MTD appears to be advantageous in developing anti-cancer drug because TU17:MTD is able to kill tumor cells in tumor tissues within 30 minutes after treatment *in vivo* (Supplementary Figure S3), which indicates that its serum stability of TU17:MTD may not be a limiting factor.

Whether pro-necrotic anti-cancer peptide can induce cell death in tumor cells that are refractory to chemotherapeutic agents and/or targeted cancer drugs is an interesting question to pursue in future. We speculate that the pro-necrotic anti-cancer peptide could kill the refractory tumor cells, based on the finding that R8:MTD killed the tumor cells that are resistant to apoptosis-inducing agents (data not shown). Thus, the pro-necrotic anti-cancer peptide would be an option to overcome the refractory tumor cells after treatment of apoptosis-inducing anti-cancer drug.

It is not clear at this point how TU17:MTD targets to NRP-1. RPAPRAR should be fused to C-term of cargo molecules based on C-end Rule described by Rouslahti's group [26]. However, the facts that the penetration and killing activity of TU17:MTD on tumor cells can be blocked by anti-NRP-1 antibody (Figures 5 and 6) indicate that TU17:MTD targets or possibly binds to NRP-1. If TU17:MTD binds to NRP-1, it could be suspected that a free arginine residue at N-term of RPARPAR behaves like a free C-term of arginine residue of C-end Rule. This possibility could be further investigated in future. Moreover, TU18:MTD (KLLNLISKLFGGRPARPAR) has the C-end Rule configuration in that MTD is conjugated to the N-term of RPARPAR, providing a free C-term of the arginine residue; however, TU18:MTD did show no or a little suppressive activity on tumor growth (Figure 1B). Another concern on TU18:MTD is that conjugation of additional amino acids at C-term of MTD reduces the killing activity of MTD. It might be the reason why TU18:MTD showed the weak suppressive activity on tumor growth compared to that of TU17:MTD.

In summary, the results of the present study suggest that pro-necrotic peptides have considerable therapeutic potential as anti-cancer drugs. We hope that the MTD-based pro-necrotic peptide represents a new platform for the development of cancer-targeting peptide drugs by combining tumor-homing motifs specific for cancer with the pro-necrotic MTD.

## MATERIALS AND METHODS

### Cell culture

All cell lines used in this study were purchased from Korean Cell Line Bank (Seoul, Korea), and were maintained by a standard culture protocol according to Korean Cell Line Bank's culture conditions.

### peptide synthesis and reagents

The peptides used in this study were synthesized from AnyGen (Gwangju, South Korea). Anti-NRP-1 antibody was purchased from Santacruz Biotechnology (Dallas, Texas, USA). Other chemicals were purchased from Sigma-Aldrich Corp. (St. Louis, MO, USA).

### Isolation of peritoneal macrophages and splenocytes

Peritoneal macrophages were isolated from mice injected intraperitoneally with 2 ml (4% (w/v) thioglycollate medium) three days prior to peritoneal lavage, which was conducted using 10 ml of RPMI 1640. Harvested cells were washed with RPMI 1640 three times and then cultured in RPMI 1640 supplemented with 10% FBS, 2 mM l-glutamine, 100 U/ml penicillin, and 100 μg/ml streptomycin. Cells were plated on culture dishes and incubated for 2 hours at 37°C in a 5% CO_2_ humidified incubator. After removing non-adherent cells, mono-layered macrophages were treated with TU17:MTD.

For splenocyte isolation, spleens were removed from BALB/c mice, placed in a Petri dish containing phosphate-buffered saline (PBS, pH 7.4), and pushed through a 200-mesh stainless steel sheet mesh. Resulting cells were suspended in PBS and centrifuged at 400 × *g* for 10 min at 20°C. Red blood cells were removed by incubation with hypotonic (0.87% (w/v) NH_4_Cl in PBS) lysis buffer for 3 minutes. Cells were then washed twice with PBS and were resuspended in RPMI 1640 medium.

### XTT assay

Cells were cultured in 96 well plates (2 × 10^5^) overnight and treated with TU:MTDs. Cell viabilities were assessed using a XTT-based colorimetric assay kit, according to the manufacturer's instructions.

### Aspartate aminotransferase (AST) and alanine aminotransferase (ALT) assays

To assay the activities of ALT and AST, mouse serum was prepared by centrifuging blood at 2500 × *g* for 15 minutes at 4°C. Enzyme activities were determined, according to the manufacturer's instructions.

### Mouse tumor models

All animal studies followed the guidelines of the Chosun University Institutional Animal Care and Use Committee. The Animal Ethics Committee at the Chosun University approved the protocols for animal experiments (Approval number: CIACUC2012-A0008, 2013-S0005 and CIACUC2015-S0019). Specific pathogen-free BALB/c mice (males, 6 weeks-old) were purchased from Samtaco (Daejon, Korea). Animals were housed under normal laboratory conditions (21–24°C and 40–60% relative humidity) under a 12-hour light/dark cycle with free access to standard rodent food and water.

Subcutaneous tumors were in BALB/c mice by injecting 1.5 × 10^5^ CT26 cells in 100 μl saline into the subcutaneous layer of the mouse. Tumor cells were grown for 7 to 8 days. TU:MTDs (approximately10 mg/kg) or saline were injected i.v. into a tail vein. Tumor volumes were calculated as length × width^2^ × 0.5 as previously described [13].

### Time-lapse confocal microscopy

For time-lapse imaging of the penetration of TU17:MTD-FAM peptides, HeLa cells or CT26 cells plated on the coverslips were placed in the chamlide magnetic chamber (Live Cell Instrument, Seoul, Korea). Cells were excited at 488 nm wavelength in the Laser Scanning Confocal Microscope (Olympus Corporation, Tokyo, Japan). Images of cells were obtained at every 1- or 2 minutes intervals for 30 minutes after addition of the indicated peptide to the medium.

### Targeting of TU17:MTD to tumor tissues

BALB/c mice harboring subcutaneous tumor tissue were injected with PBS, TU17:MTD-FAM, or TU17:MTD-FAM plus TU17:ΔMTD. One hour upon peptide injection, the mice were sacrificed to harvest tumor tissues and blood samples. The fluorescence intensities of tumor tissues were measured by Xenogen IVIS 200 imaging system, and were quantified by using a living imaging software (Caliper Life Sciences, Hopkinton, MA, United States). The fluorescence intensities of blood samples were measured using Sunrise microplate reader (Tecan, Mannedorf, Switzerland).

### Statistical analysis

Results are reported as means ± SDs. ANOVA test was used to evaluate differences between more than two groups. When a significant difference was observed, Dunnet's ‘*t*’ test was used to compare the means of two groups. Statistical significance was accepted for *p* values < 0.05.

## SUPPLEMENTARY MATERIAL FIGURES AND TABLES


