# Correction: Necrosis-inducing peptide has the beneficial effect on killing tumor cells through neuropilin (NRP-1) targeting

**DOI:** 10.18632/oncotarget.25604

**Published:** 2018-06-01

**Authors:** Ji-Young Kim, Ji-Hae Han, Geon Park, Young-Woo Seo, Cheol-Won Yun, Byung-Chul Lee, Jeehyeon Bae, Ae Ran Moon, Tae-Hyoung Kim

**Affiliations:** ^1^ Department of Biochemistry, Chosun University School of Medicine, Dong-Gu, Gwang-Ju, Korea; ^2^ Department of Laboratory Medicine, Chosun University School of Medicine, Dong-Gu, Gwang-Ju, Korea; ^3^ Korea Basic Science Institute Gwang-Ju Center, Chonnam National University, Buk-Gu, Gwang-Ju, Korea; ^4^ School of Life Science and Biotechnology, Korea University, Seoul, Korea; ^5^ Genoflux. Co., Ltd., Sejong City, Korea; ^6^ School of Pharmacy, Chung-Ang University, Dongjak-Gu, Seoul, Korea

**This article has been corrected:** The correct Grant Support information is given below:

**GRANT SUPPORT**

This research was supported by the Basic Science Research Program through the National Research Foundation of Korea funded by the Ministry of Education, Science and Technology (Grant no. 2010-0009201 and NRF-2013R1A1A2058254 to TH Kim), by the Ministry of Science, ICT, and Future Planning (NRF-2014R1A2A1A11050442 to TH Kim), and by the National R&D Program for Cancer Control funded by the Ministry of Health and Welfare of the Korean government (Grant no. HI14C2025 to TH Kim).

Original article: Oncotarget. 2016; 7:32449-32461. https://doi.org/10.18632/oncotarget.8719

