# Supplementary material for: Necrosis-inducing peptide has the beneficial effect on killing tumor cells through neuropilin (NRP-1) targeting
Source: Oncotarget. 2016 Apr 13;7(22):32449–61. doi: 10.18632/oncotarget.8719 (PMC5078025; doi:10.18632/oncotarget.8719)
Supplement: Supplementary file 1 [file oncotarget-07-32449-s001.pdf]

Necrosis-inducing peptide has the beneficial effect on killing tumor cells through neuropilin (NRP-1) targeting

Supplementary Materials

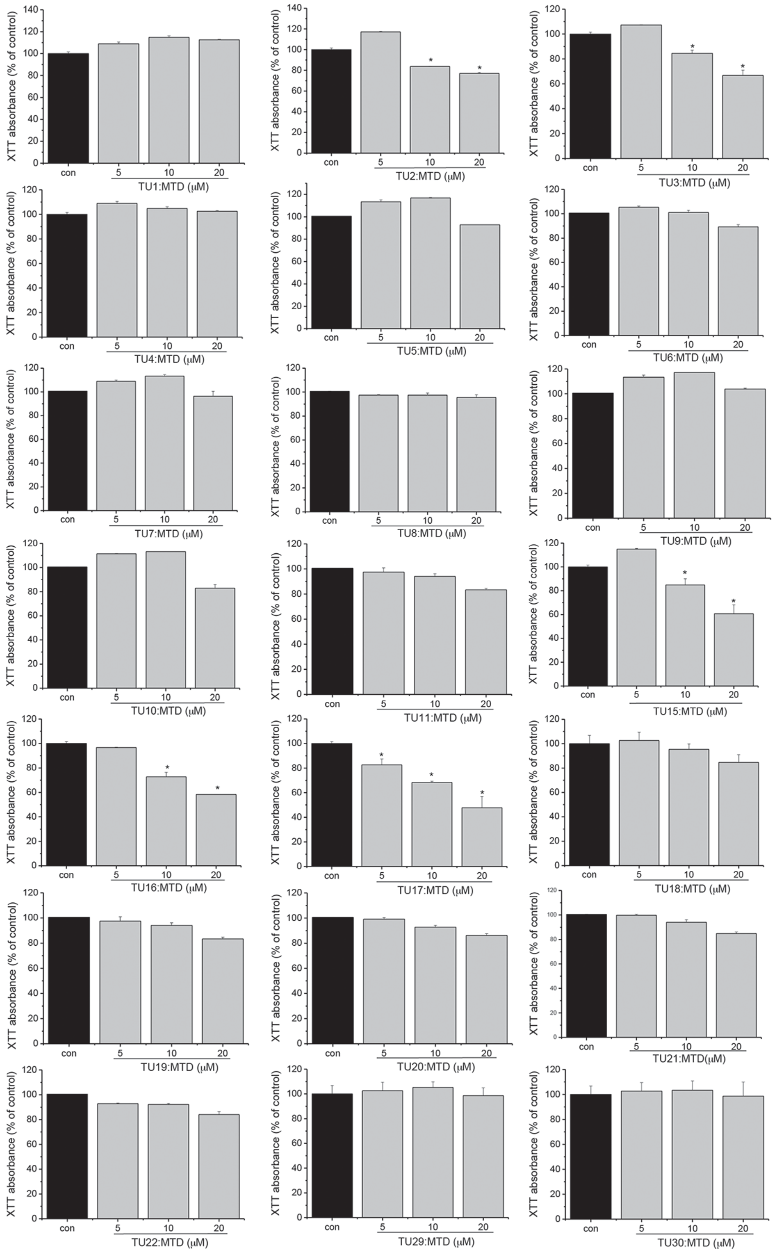

Supplementary Figure S1: Killing activity of TU:MTDs in CT26 cells. CT26 cells were treated with TU:MTDs (0 ~ 20 μM) for 30 minutes, and cell viability was monitored using XTT assays. Results are expressed as means ± SD, and are representative of at least two independent experiments. *$P < 0.05$.

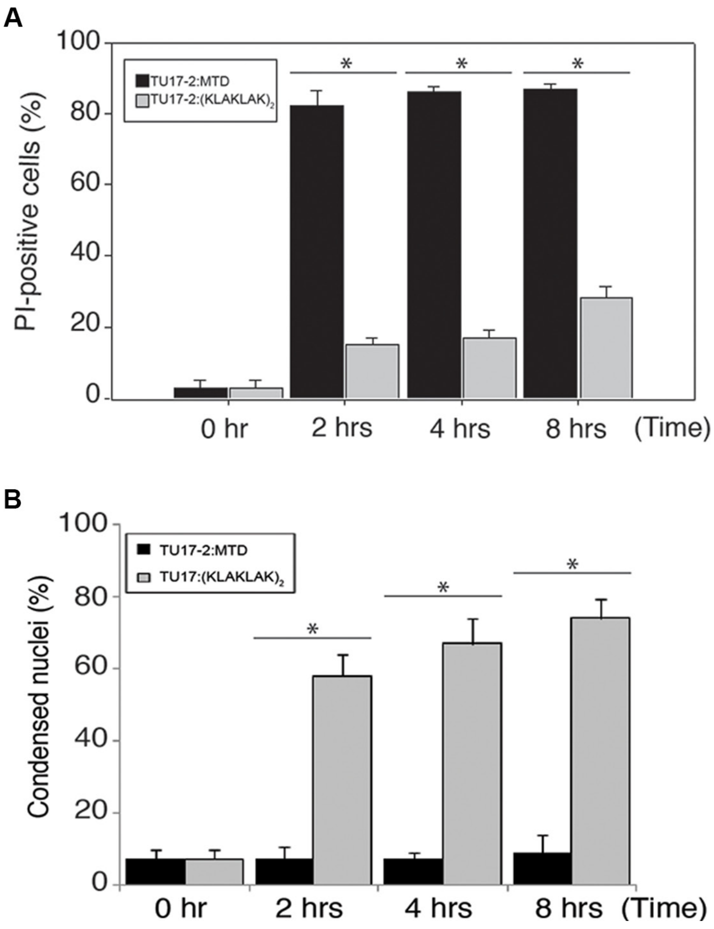

Supplementary Figure S2: TU17-2:MTD and TU17-2:(KLAKLAK)$_2$ cause necrosis and apoptosis, respectively, in CT26 cells. CT26 cells were treated with TU17-2:(KLAKLAK)$_2$ or TU17-2:MTD, and were stained with Hoechst/PI. Images were obtained using an Olympus confocal microscope. PI-positive cells (**A**) or cells with condensed nuclei (**B**) were counted (n = over 200 cells). *$P < 0.05$.

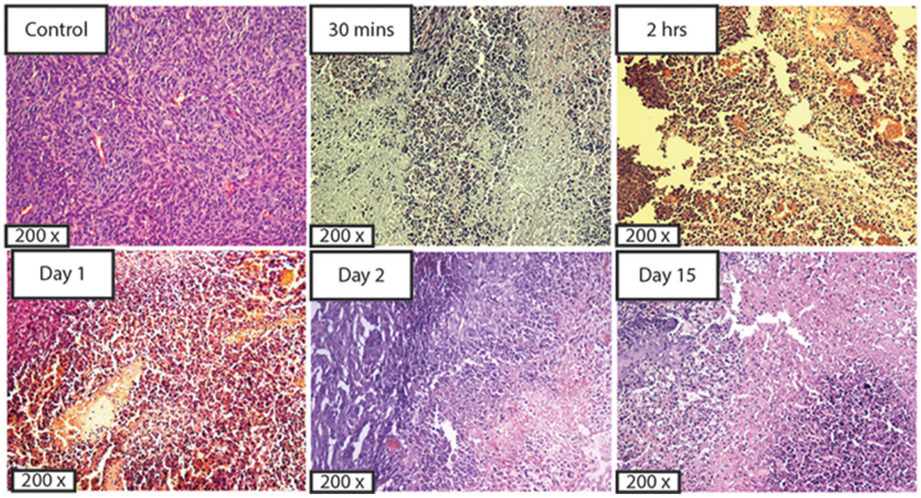

Supplementary Figure S3: Effect of TU:MTDs on subcutaneous tumor bearing CT26 cells in BALB/c mice. (**A**) Tumors were generated over 2 weeks after s.c. injection of CT26 cells into the back of BALB/c mice. When tumor volumes reached around 90 ± 10 mm^3, TU17-2:MTD (250 µg/mouse/day), TU17-2:ΔMTD (190 µg/mouse/day) or PBS were administrated i.v. Tumor dimensions in mice from the TU:MTDs-treated groups (n = 7) and PBS-treated group (n = 7) were measured with a caliper and tumor volumes were calculated using longest diameter × width^2 × 0.5 at the indicated days. (**B**) Experiments were performed as described in Figure 4A. Tumor tissues obtained from TU17:MTD-treated mice at the indicated time points or from PBS-treated mice at day 0 as a control were stained with H&E, and images (Magnification; 200 × and 400 ×) were taken under a light microscope. Arrows indicate lymphocytes at day 15.

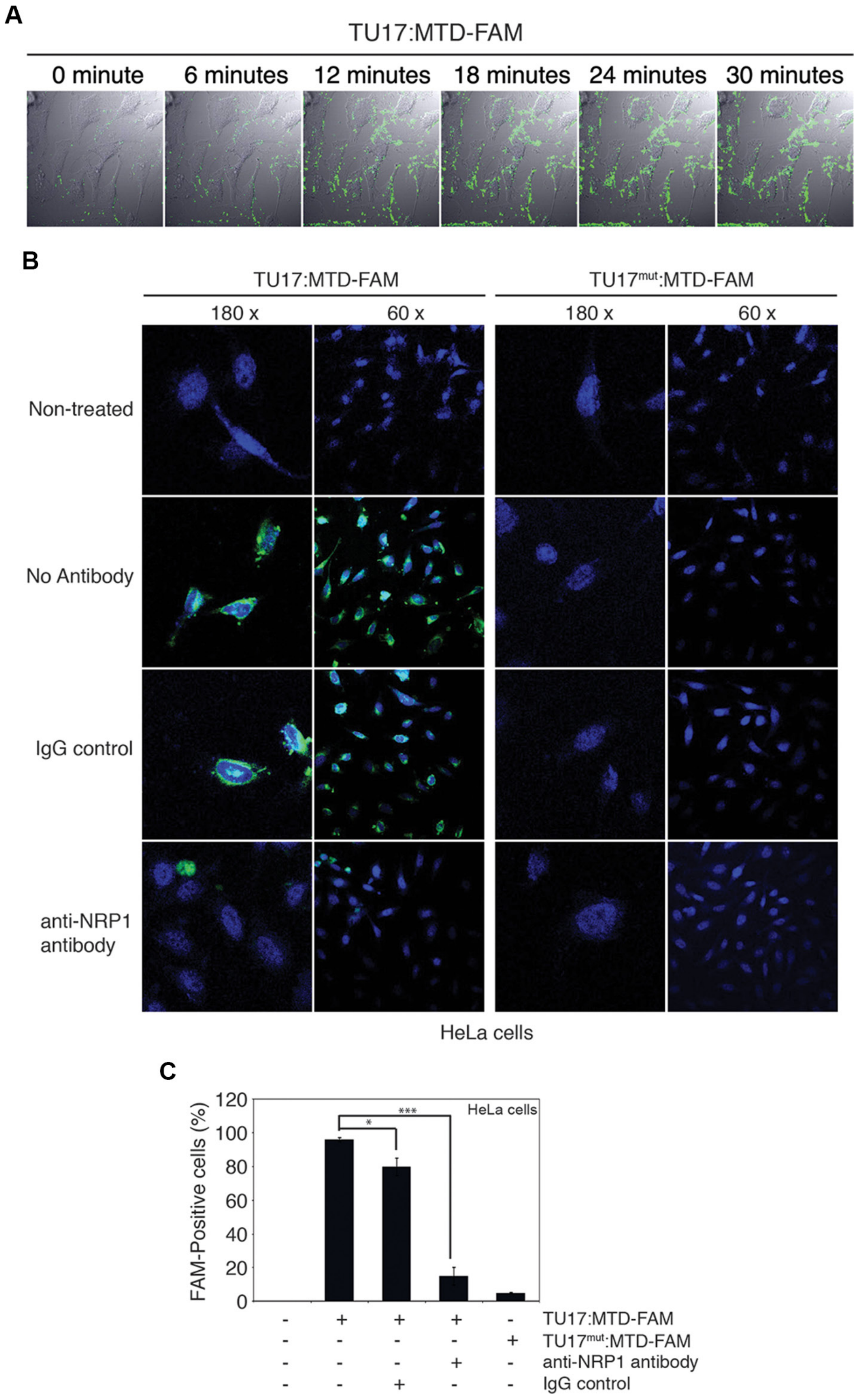

Supplementary Figure S4: Anti-NRP-1 antibody blocks the entry of TU17:MTD-FAM into tumor cells. (**A**) HeLa cells (2×10^4 cells/cm^2) were treated with TU17:MTD-FAM (500 nM) peptide. Live cell images (magnification 60 ×) were obtained at 6-minute intervals for 30 minutes using an Olympus confocal microscope. (**B**) HeLa cells were treated with peptides as mentioned in Figure 5C. The images were captured at 30 minutes after treatment with Olympus confocal microscope. (**C**) The experiments were performed as described in Figure 5C. The percentage of FAM-positive cells (%) were determined (*n* = over 200 cells). $*P < 0.05$, $***P < 0.001$

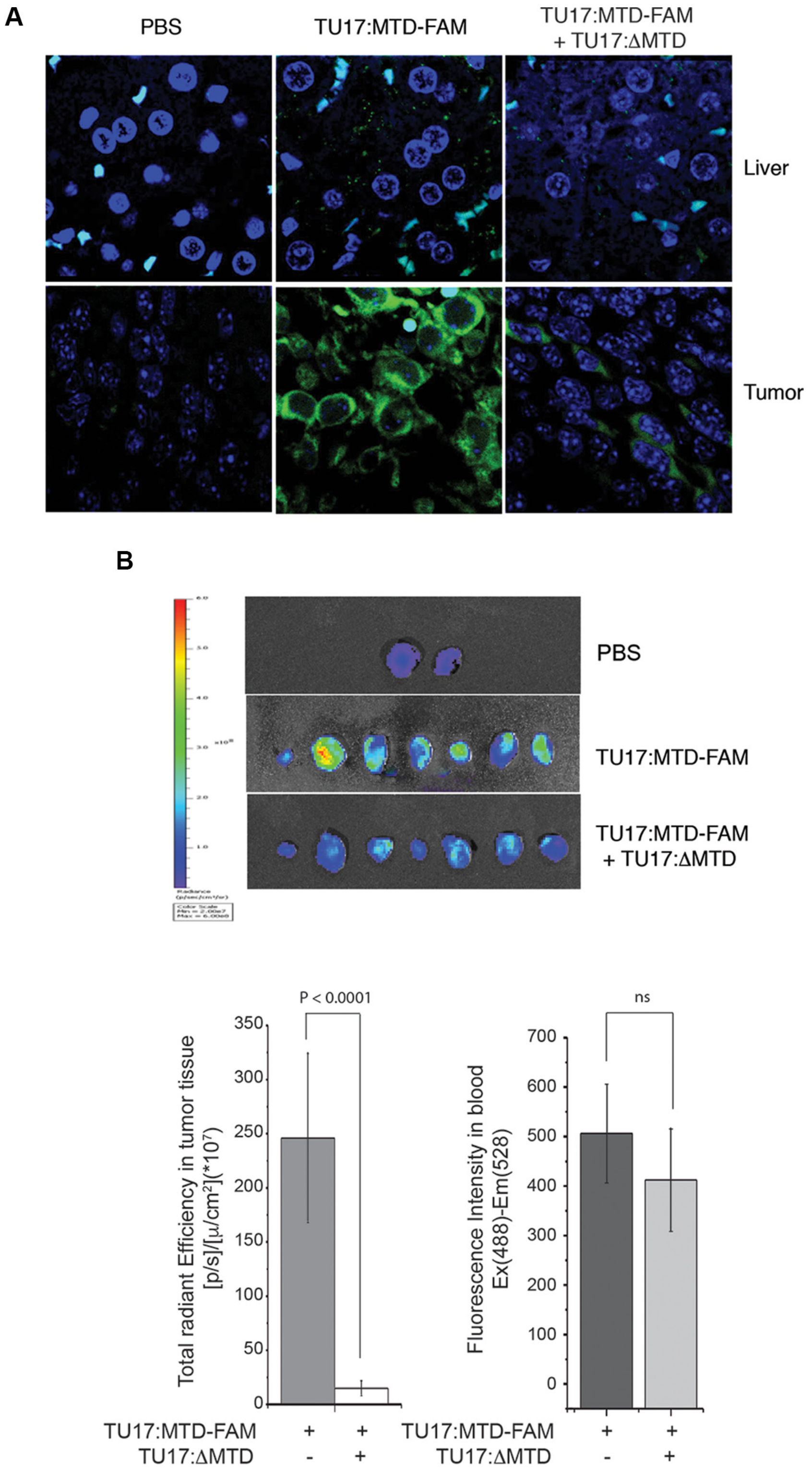

Supplementary Figure S5: ATargeting of TU17:MTD to tumor tissue. (**A**) PBS alone (*n* = 2), TU17:MTD-FAM (100 μl of 1 mM, *n* = 7), or TU17:MTD-FAM plus TU17:ΔMTD (100 μl of 1 mM TU17:MTD-FAM plus 100 μl of 1 mM TU17: ΔMTD, *n* = 7) were i.v. injected into mice bearing CT26 tumors. At one hour after injection, the mice were sacrificed, and tumor tissues (magnification 400 ×) were processed to detect fluorescence using a confocal microscope after counterstaining with DAPI. (**B**) BALB/c mice bearing tumor were i.v. injected with PBS alone (*n* = 2), TU17:MTD-FAM (100 μl of 1 mM, *n* = 7), or TU17:MTD-FAM plus TU17:ΔMTD (100 μl of 1 mM TU17:MTD-FAM plus 100 μl of 1 mM TU17: ΔMTD, *n* = 7). At one hour after injection, the mice were sacrificed to obtain tumor tissues. The fluorescence intensities of tumor tissues were measured by Xenogen IVIS 200 imaging system, and were quantified by using a living imaging software (Caliper Life Sciences, Hopkinton, MA, United States). Blood from mice injected TU17:MTD-FAM (*n* = 7), or TU17:MTD-FAM plus TU17:ΔMTD (*n* = 7) as described above was harvested, and the fluorescence intensities were measured using Sunrise microplate reader (Tecan, Mannedorf, Switzerland). NS (non-signficant).

Supplementary Table S1: Sequences of TU:MTDs

Names	Sequences	Type
TU1:MTD	CNGRCGGKLLNLISKLF	Linear
TU2:MTD	CNGRCVSGCAGRCGGKLLNLISKLF	Linear
TU3:MTD	CGNKRTRGCGGKLLNLISKLF	Linear
TU4:MTD	WIFPWIQLKLLNLISKLF	Linear
TU5:MTD	WDLAWMFRLPVGKLLNLISKLF	Linear
TU6:MTD	CGRDKGPDCKLLNLISKLF	Linear
TU7:MTD	KLLNLISKLFCGRDKGPDC	Linear
TU8:MTD	KLLNLISKLFCGRDKRLYDC	Linear
TU9:MTD	CRGDKGPDCKLLNLISKLF	Linear
TU10:MTD	KLLNLISKLFCRGDKGPDC	Cyclic
TU11:MTD	KLLNLISKLFCRGDKRLYDC	Cyclic
TU15:MTD	RLLRLLRLLRGGKLLNLISKLF	Linear
TU16:MTD	KLLNLISKLFGGRLLRLLRLLR	Linear
TU17:MTD	RPARPARGGKLLNLISKLF	Linear
TU18:MTD	KLLNLISKLFGGRPARPAR	Linear
TU19:MTD	CGKRKGGKLLNLISKLF	Linear
TU20:MTD	KLLNLISKLFGGCGKRK	Linear
TU21:MTD	CRGDKGGKLLNLISKLF	Linear
TU22:MTD	KLLNLISKLFGGCRGDK	Linear
TU29:MTD	RGDRGDRLLRGGKLLNLISKLF	Linear
TU30:MTD	KLLNLISKLFGGRGDRGDRLLR	Linear

TUs indicate tumor homing motifs, and MTD refers to mitochondrial targeting domain of Noxa.

Supplementary Table S2: Toxicity of TU:MTDs and activity of TU:MTDs on tumor growth

Names	Toxicity/Tumor growth	Movement Activities
TU1:MTD	NR	Slow
TU2:MTD	NR	Normal
TU3:MTD	NR	Normal
TU4:MTD	Toxic	Slow
TU5:MTD	ND	Normal
TU6:MTD	ND	Normal
TU7:MTD	ND	Linear
TU8:MTD	Dead	Dead
TU9:MTD	ND	Normal
TU10:MTD	ND	Slow
TU11:MTD	Toxic	Slow
TU15:MTD	Toxic	Slow
TU16:MTD	Toxic	Normal
TU17:MTD	Tumor regression (1 out of 3 mice)	Normal
TU18:MTD	NR	Slow
TU19:MTD	NR	Normal
TU20:MTD	NR	Normal

TU21:MTD	NR	Slow
TU22:MTD	NR	Normal
TU29:MTD	ND	ND
TU30:MTD	NR	ND

BALB/c mice bearing CT26 tumor were i.v. injected with the indicated TU:MTDs (75 µl of 1 mM TU:MTDs) twice or three times. Tumor growth in the TU:MTDs-treated groups (*n* = 3) and PBS-treated group (*n* = 3) were observed until 9 days since the first injection of the peptides. Most of tumors injected with TU:MTDs were not regressed, and some TU:MTDs caused necrosis in tail vein upon injection. Movement activities were observed within 30 minutes after TU:MTD peptides (75 µl of 1 mM) were i.v. injected into BALB/c (around 20 gm) mice at a single dose. “Slow” means not moving around in the cage. “Normal” means normally moving around in the cage. “Toxic” means necrosis in tail vein within 2 ~ 3 days after injection. Abbreviation: NR; no regression, ND; not determined.

Supplementary Table S3: Serum levels of AST (aspartate aminotransferase) and ALT (alanine aminotransferase) in mice treated with TU17:MTD

Treatment	Serum aminotransferase (karman units/ml)	
	AST	ALT
Normal control	5.9 ± 2.1	40.1 ± 2.2
CCl_4	42.8 ± 3.7	558.8 ± 7.2
PBS control (tumor)	9.2 ± 2.3	42.8 ± 4.1
TU17:MTD (30 mins)	8.1 ± 0.5	38.5 ± 2.3
TU17:MTD (2 hours)	8.4 ± 2.1	38.2 ± 5.4
TU17:MTD (day 1)	9.8 ± 1.2	39.1 ± 4.5
TU17:MTD (day 2)	7.4 ± 1.1	37.5 ± 5.8
TU17:MTD (day 4)	7.1 ± 1.9	35.2 ± 2.7
TU17:MTD (day 8)	7.1 ± 1.5	38.3 ± 3.5
TU17:MTD (day 15)	7.1 ± 1.5	40.2 ± 2.1

Supplementary Table S4: Complete blood counting of mice treated with TU17:MTD or TU17:MTD^{4A}

Treatments	White blood cells (×10^3 cell/µl)	Hemoglobin (g/dl)	Platelets (×10^3 cell/µl)	Neutrophils (%)	Lymphocyts (%)	Monocytes (%)
Normal (w/o tumor)	0.90 ± 0.26	4.17 ± 0.32	272.3 ± 21.36	23.33 ± 2.5	70.33 ± 5.77	6.33 ± 4.04
Control (w/ tumor)	1.08 ± 0.55	3.90 ± 0.52	478.33 ± 118.98	53 ± 7.54	38.66 ± 5.5	8.33 ± 3.78
TU17:MTD (30 mins)	1.43 ± 0.36	4.60 ± 0.14	140.50 ± 34.65	36.5 ± 14.84	42.17 ± 16.26	3.26 ± 1.41
TU17:MTD^{4A} (30 mins)	1.78 ± 0.27	4.60 ± 0.28	277.00 ± 46.67	46 ± 11.31	35.42 ± 2.82	6.47 ± 8.48
TU17:MTD (2 hrs)	1.16 ± 0.44	4.05 ± 0.35	322.50 ± 61.52	51.5 ± 7.78	32.28 ± 5.65	3.83 ± 2.12
TU17:MTD^{4A} (2 hrs)	1.09 ± 0.03	4.40 ± 0.28	287.50 ± 14.85	52 ± 15.56	31.55 ± 13.44	3.04 ± 2.12
TU17:MTD (day 1)	1.61 ± 0.78	3.93 ± 0.51	250.33 ± 68.97	67.5 ± 13.44	30 ± 14.14	2.5 ± 0.71
TU17:MTD^{4A} (day 1)	0.79 ± 0.37	4.40 ± 0.30	327.00 ± 30.51	32 ± 26.21	66 ± 26.23	2 ± 2.65

Four leucine residues in TU17:MTD (RPARPARGGK*LL*N*L*ISK*L*F) is replaced by four alanines in TU17:MTD^{4A} (RPARPARGGK*AA*N*A*ISK*A*F).
